# Beyond vision: effects of light on the circadian clock and mood-related behaviours

**DOI:** 10.1038/s44323-025-00029-1

**Published:** 2025-03-13

**Authors:** Dean Stewart, Urs Albrecht

**Affiliations:** Department of Biology, University of Fribourg, Fribourg, Switzerland

**Keywords:** Circadian rhythms and sleep, Neuroscience, Diseases, Psychiatric disorders, Depression

## Abstract

Light is a crucial environmental factor that influences various aspects of life, including physiological and psychological processes. While light is well-known for its role in enabling humans and other animals to perceive their surroundings, its influence extends beyond vision. Importantly, light affects our internal time-keeping system, the circadian clock, which regulates daily rhythms of biochemical and physiological processes, ultimately impacting mood and behaviour. The 24-h availability of light can have profound effects on our well-being, both physically and mentally, as seen in cases of jet lag and shift work. This review summarizes the intricate relationships between light, the circadian clock, and mood-related behaviours, exploring the underlying mechanisms and its implications for health.

## Introduction

Light perceived by the eyes passes through the lens and hits the retina at the back of the eye, leading to the formation of images and enabling the tracking of objects^1^. Importantly, the eyes communicate information regarding light not only for vision but also to regulate various physiological and behavioural functions, many of which are independent of image formation, termed non-image-forming functions. These include the adjustment of the circadian clock to environmental light, suppression of sleep, and changes in alertness and mood. Hence, light is vital for both image formation and non-image-forming functions^2^.

As the Earth rotates on its axis and around the Sun, light is not constantly present (except in polar regions during summer or winter seasons, respectively). However, due to the geophysical properties of the Earth, the change in lighting conditions is predictable. This predictability allowed organisms on Earth to develop a mechanism in the form of an internal clock with a period of about one day (circa diem) that can track and anticipate environmental changes i.e., day-night transitions but also seasonal changes in day length (photoperiod). This circadian clock is self-sustaining and continues to cycle even in the absence of light; however, it can adjust its phase according to environmental lighting^3^. Within an organism, the clock regulates biochemical and physiological processes, aligning metabolism and behaviour accordingly. Consequently, body function is synchronized with environmental demands^4^. This temporal alignment between light, the clock, and physiological processes is beneficial for an organism’s survival in a competitive environment^5,6^.

One of the core symptoms in mood disorders, including seasonal affective disorder (SAD)^7^, major depressive disorder (MDD)^8–10^, and bipolar disorder (BP)^11,12^ is abnormal sleep/wake patterns, which serve as one of the diagnostic criteria for mood disorders^11^. Sleep is a fundamental state of quiescence with reduced mental and physical activity, altered consciousness, and inhibited sensory activity. It is regulated on one hand by circadian clock mechanisms that determine sleep timing, and on the other hand by a homoeostatic component that determines sleep drive, which can override circadian timing^13^. Importantly, light can influence both circadian and homoeostatic components of sleep^14–17^ and it suppresses secretion of melatonin, a molecule with a multitude of physiological actions^18^. However, it is unclear whether sleep disturbances are the cause or consequence of mood disorders.

In this review, we discuss studies in mice that describe the non-image-forming functions of light that affect the circadian clock and mood-related behaviours at the molecular level. The extension of these findings in mice to human disease relevance is also discussed.

## How light reaches the brain

**Fig. 1:**
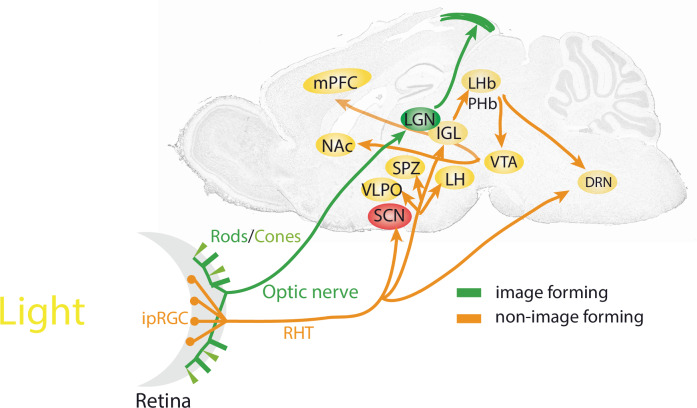
Retinal brain connections underlying the effects of light on visual functions. Image-forming visual functions of rods and cones projecting to the LGN and the tectum are shown in green. IpRGCs project via the RHT (orange) to various brain areas for non-image-forming visual functions such as photoentrainment and mood-related behaviours. DRN dorsal raphe nuclei, IGL intergeniculate leaflet, ipRGC intrinsically photosensitive retinal ganglion cell, LGN lateral geniculate nucleus, LH lateral hypothalamus, LHb lateral habenula, mPFC medial prefrontal cortex, NAc nucleus accumbens, PHb perihabenula, RHT retinohypothalamic tract, SCN suprachiasmatic nuclei, SPZ subparaventricular zone, VLPO ventrolateral preoptic area, VTA ventral tegmental area.

Image formation begins when photons of light hit photoreceptors (rods and cones) lining the retina at the back of the eye. Information regarding light is communicated via the optic chiasm to the lateral geniculate nucleus (LGN) and then the occipital lobe visual cortex (Fig. 1), where image processing takes place. Cones are primarily responsible for colour vision, functioning best in bright light (i.e., during the day), while rods are specialized for the detection of low light levels, enabling organisms to see during dawn and dusk^19^.

In recent years, non-image-forming pathways by which light influences the brain have been identified in the mouse. Intrinsically photosensitive retinal ganglion cells (ipRGCs) containing the photopigment melanopsin^20^, send glutamatergic projections via the retino-hypothalamic tract (RHT; Fig. 1) towards the mammalian master clock, the suprachiasmatic nuclei (SCN)^21^. Here, the phase of the circadian clock is shifted by nocturnal light directly^22^ while processes such as hippocampal learning are located downstream and thus indirectly affected by light^23^. Furthermore, various subtypes of ipRGCs project to the subparaventricular zone (SPZ) and intergeniculate leaflet (IGL), both of which are important for clock regulation, as mediators of SCN signals and as participants in broader networks of circadian regulation. Other targets include areas involved in sleep regulation such as the ventrolateral preoptic area (VLPO) and lateral hypothalamus (LH), or the medial amygdala and lateral habenula (LHb), which are implicated in the regulation of mood^24^. Recent studies show that ipRGCs encoding light-intensity innervate the perihabenula (PHb). The PHb in turn projects to the LHb, affecting the ventral tegmental area (VTA), the nucleus accumbens (NAc) and the medial prefronatal cortex (mPFC), driving light-mediated alterations in mood (Fig. 1)^23,25–27^. ipRGC-like properties which encode changes in ambient lighting levels (irradiance) have also been detected in human prefrontal regions revealing light-intensity-dependent responses, suggesting that the pathway by which light affects mood and cognition in mice may also exist in humans^28^. Interestingly, a retino-raphe pathway, a direct connection between the retina and the dorsal raphe nucleus (DRN) has been described in the cat^29^. ipRGCs as well as other types of RGCs project to the DRN^30^ to regulate serotonergic and GABAergic activity^31^ ultimately affecting depression-like behaviour^32^. This pathway may also exist in humans as indirectly evidenced by variation of serotonin levels in blood samples collected in different seasons and lighting conditions^33^. However, the molecular details of how light may affect serotonin and GABA levels in the DRN are unknown.

Melatonin, a modulatory hormone produced by the pineal gland is inhibited by light^34,35^, enabling the encoding and communication of day length (photoperiod) across seasons and may play minor contrasting roles in sleep vs activity in diurnal^36^ and nocturnal mammals^37^, respectively. For an in-depth review for melatonin in circadian rhythms and sleep see ref. ^18^. The importance of ipRGCs in non-image forming processes is evident in visually blind humans, who lack rods and cones but not ipRGCs, and can still detect light and inhibit the secretion of melatonin^34^. Furthermore, mice lacking rods and cones can still align to the day-night cycle (termed photoentrainment)^15,38–40^. Interestingly, however, mice which lack intrinsically photosensitive ipRGCs due to the loss of the photopigment melanopsin show slightly attenuated photoentrainment (but also in non-image forming responses i.e., pupil constriction) in response to monochromatic (one colour) light, however, as clock phase-shifting responses are not completely abolished this indicates that other photoreceptors (i.e., rods and cones) play a role in photoentrainment. While changes in light levels during the day are prominent, another change that occurs is the spectral (colour) composition of light, with the ratio of blue-to-yellow colour shifting as the sun sets^41^. Notably, the absence of this change in blue-to-yellow ratio shifted rodent body temperature and SCN electrical activity to earlier time points^42^, indicating a role for cones in photoentrainment. Importantly, mice lacking all three photoreceptors cannot align their activity to the day-night cycle^43^. Surprisingly, the complete loss of ipRGCs produces this same phenotype^44^, indicating a central role of ipRGCs in communicating non-image-forming light responses by relaying extrinsic information about light from rods and cones but also intrinsic light information coming from the pigment melanopsin^45^.

## Light and the circadian clock

**Fig. 2:**
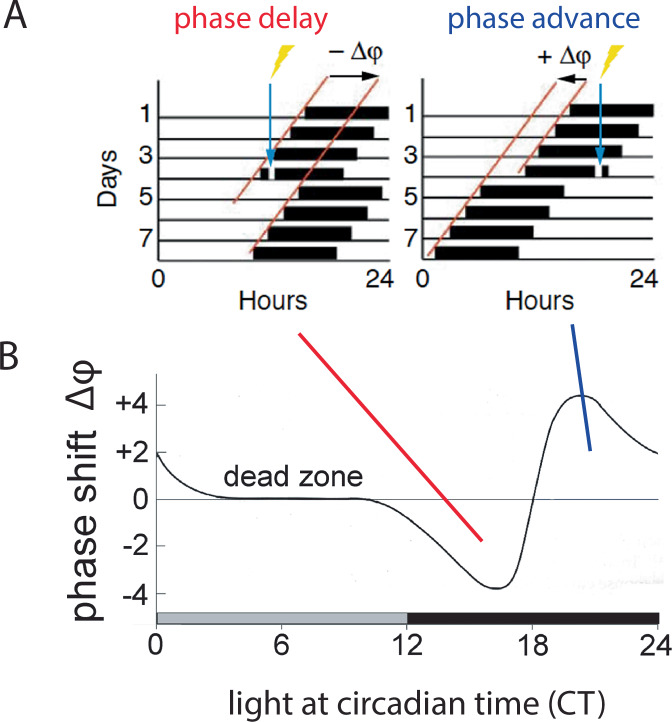
The Phase Response Curve (PRC). **A** Brief light exposure during the early subjective night (blue arrow, left panel) will delay the onset of the subsequent activity cycle, resulting in a phase delay of the clock characterized by a negative Δφ (phase angle difference of the two red lines of activity onset). By contrast, light applied in the late portion of the subjective night (blue arrow, right panel) induces phase advances (positive Δφ). This change in phase can be plotted as a function of time when light is applied, resulting in the phase response curve (**B**), which serves as a read-out for how light entrains (resets) the clock.

Environmental lighting aligns the internal circadian clock and the sleep-wake cycle to the solar day. This defines the organism’s activity to the correct temporal niche, either during the day (diurnal) or during the night (nocturnal). Hence, light acts as a time-giver (Zeitgeber) to the SCN (Fig. 1)^46^, aligning the circadian clock, whose period length differs slightly from 24 h, to the day-night cycle. Artificially induced light changes, as experienced during shift-work or as the results of jet-lag, can directly affect the SCN molecular clock. Temporal changes in light levels induce adaptive behaviours, either advancing or delaying an organism’s activity phase relative to when the light is presented, as illustrated in the phase-response curve to light (Fig. 2)^47,48^. Hence, light late in the dark period (i.e., night time) advances clock phase, whereas light in the early part of the dark period delays it.

**Fig. 3:**
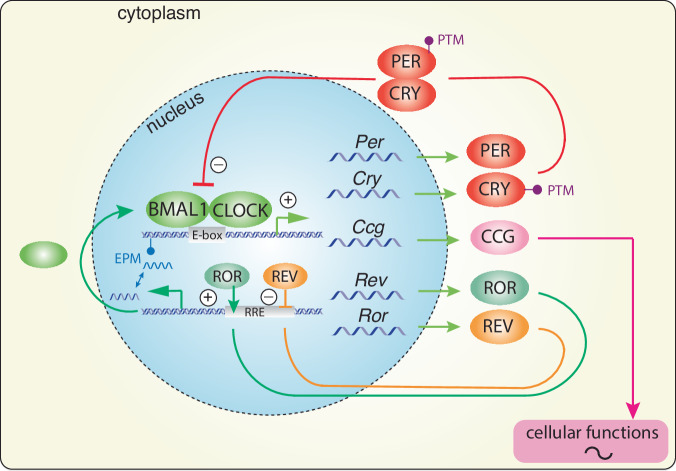
Cellular and molecular mechanism of the circadian clock. Brain and muscle ARNT-like protein (BMAL1) and circadian locomotor output cycles kaput (CLOCK) heterodimerize and bind to enhancer box (E-box) elements in the promoters of clock-controlled genes (*Ccgs*) and clock genes *Period* (*Per*), *Cryptochrome* (*Cry*), *retinoid orphan receptor* (*Ror*), and *reverse strand of c-erbA* (*Rev-erb*). PER and CRY proteins dimerize and translocate back to the nucleus to inhibit BMAL1/CLOCK activity, forming a transcriptional/translational negative feedback loop (TTL) that cycles about every 24 h. In a secondary loop, the nuclear hormone receptors ROR and REV-ERB activate or inhibit *Bmal1* transcription, respectively. Post-translational modifications (PTMs) determine the stability and/or nuclear localization potential of clock components, thereby influencing the speed of the TTL. Epigenetic modifications (EPMs) influence the TTL at the genetic level.

How exactly circadian behaviour is linked to molecular events has been an intensely researched topic. The first convincing evidence demonstrating the governance of the circadian clock by genetic mechanisms was carried out in chemically mutated fruit flies, which lacked circadian activity behaviour in constant darkness^49^. The cloning of specific genes in the fly and the demonstration of an autoregulatory feedback loop as the basis of a circa 24-hour oscillation in the behavioural activity of the fly^50–52^ led to the elucidation of the underlying clock mechanism in mammals^53^. Like in the fly, the mammalian circadian clock mechanism consists of transcriptional and translational processes that generate a cell-autonomous autoregulatory feedback-loop with a period of ~24 h. The core clock genes are *Bmal1* and *Clock* (and its homologue *Npas2*), which bind to specific promoter elements called E-boxes, activating transcription of *Per* and *Cry* genes. PER and CRY proteins, in turn, repress their own transcription by inhibiting BMAL1 and CLOCK complexes (Fig. 3). This, combined with the activity of the nuclear receptors REV-ERBα and RORα, regulates the expression of clock components *Bmal1*, *Clock* and *Npas2*, repressing or activating their transcription, respectively, ultimately generating the clock mechanism with a periodicity of ~24 h^54^ (Fig. 3). The period of the clock is fine-tuned by many additional levels of regulation, including the modification of DNA (epigenetic mechanisms, EPM) through methylation, acetylation, and non-coding RNA intervention, or the modifications of clock proteins themselves (post-translation modification; PTM) e.g. with phosphorylation and sumoylation^55,56^. Importantly, phosphokinases, which drive the proteasomal degradation of PER proteins through phosphorylation^57^, may provide a mechanism to couple external stimuli (e.g., light) with the clock. Indeed, mice lacking protein kinase-C have defects in light-induced resetting of the clock^58^.

The timing information generated by this autoregulatory feedback-loop can align important molecular processes within the organism. Indeed, many genes encoding rate-limiting enzymes or those involved in the regulation of key physiological processes contain E-box promoter elements; hence, they are activated in a clock- dependent fashion and can be referred to as clock-controlled genes (*Ccgs;* Fig. 3). Thus, important biological processes are temporally controlled, including metabolism and neurotransmitter expression. Consequently, the peak activity of these processes is aligned appropriately to the time of day when their function is most crucial^59^. Hence, the output of the circadian clock is the regulation of physiology and behaviour.

**Fig. 4:**
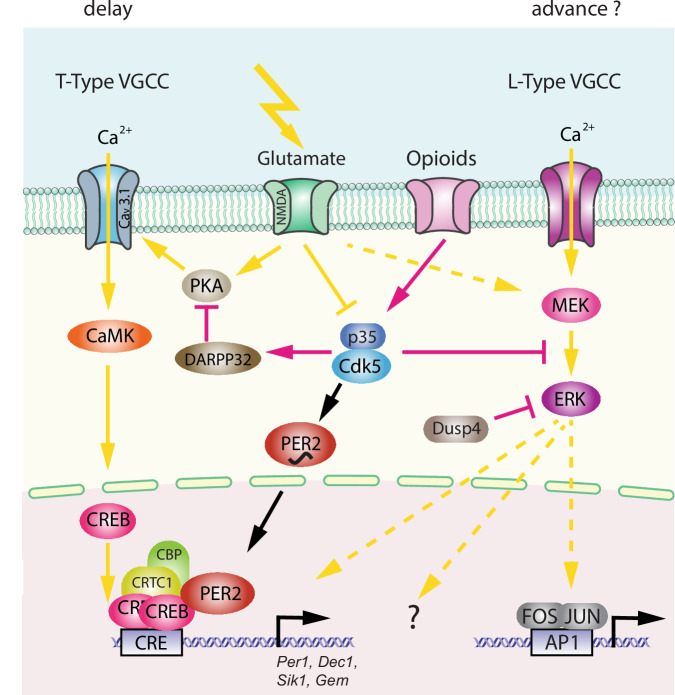
Molecular model of light-induced signalling pathways for clock phase shifting. The left part of the model depicts the phase delay process involving T-type voltage-gated calcium channels (VGCCs), and the right part illustrates a hypothetical phase advance process involving L-type VGCCs. The delay model involves activating glutamate signalling via protein kinase A (PKA), Cav 3.1, Ca^2+^/calmodulin-dependent protein kinase (CaMK), and cAMP response element-binding protein (CREB), which binds to CRE promoter elements of target genes such as *Per1*, *Dec1*, *Sik1*, and *Gem*. This transcriptional activation is supported by co-factors such as CREB-regulated transcription coactivator 1 (CRTC1), CREB-binding protein (CBP), and PER2. PER2 and PKA are regulated by Cdk5, whose activity depends on interaction with p35/25, which is influenced by light and opioids. The phase advance mechanism is unclear (hatched arrows) and may involve the MEK/ERK signalling pathway, which may also influence phase delays and FOS/JUN transcriptional activation.

As described above, circadian activity behaviour is modulated by light. Since behaviour is regulated by the clock, behaviour must be altered by light. IpRGCs activated by light promote the release of several neurotransmitters involved in photic stimulation from the RHT at target brain regions, including glutamate, pituitary adenylate cyclase-activating polypeptide (PACAP) and substance P. Glutamate from the RHT generates an influx of Ca^2+^ into SCN cells by activating N-methyl-D-aspartate (NMDA) and α-amino-3-hydroxy-5-methyl-4-isoxazolepropionic acid (AMPA) receptors^60^, ultimately activating several intracellular signalling pathways. This involves changes in activity of various kinases, including cyclin-dependent kinase 5 (CDK5), dopamine and cAMP-regulated phosphoprotein of 32 kD (DARPP-32), protein kinase A (PKA), and Ca^2+^/calmodulin-dependent kinases (CaMK), culminating in the phosphorylation of cyclic AMP response element binding protein (CREB) (Fig. 4)^61–66^.

This promotes phosphorylation^67^ and acetylation of chromatin by recruiting cAMP-regulated transcriptional co-activator 1 (CRTC1) and the histone acetyltransferase CREB-binding protein (CBP), which involves the clock protein PER2^68^. Consequently, immediate-early genes such as *cFos* and clock genes such as *Per1* are induced (Fig. 4)^69–71^, causing a rapid shift in the phase of the clock autoregulatory feedback loop in oscillating SCN cells^72^. This manifests at the behavioural level in delaying activity onset (Fig. 2, between CT12-18). Furthermore, CRTC1 is expressed rhythmically in the SCN, peaking during the middle of the day and light during the early or late night induces strong nuclear accumulation of CRTC1, leading to the induction of salt-inducible kinase 1 (SIK1) in addition to *Per1* (as mentioned above^73,74^). Negative regulation of CREB occurs via SIK1 through the phosphorylation and inactivation of CRTC1, forming a negative autoregulation feedback loop. Importantly, SCN knockdown of SIK1 enhances phase-shifting responses to a phase-delaying light pulse and enables rapid re-entrainment to a shift in the light cycle i.e., as experienced in jet lag^74^. The advancement of activity onset (phase advance as seen in Fig. 2, between CT18-24), however, involves another signalling pathway that is not yet fully understood, except for the observation that L-type voltage gated calcium channels (L-type VGCCs) are important^75,76^. Additionally, cholecystokinin-expressing neurons may play a role as their specific activation induced phase advances^77^. Taken together, it appears that light signals perceived in the early portion of the dark phase elicit rapid phase delays via CREB signalling, PER proteins, and T-type VGCCs^66,75^, whereas light perceived in the late part of the dark phase elicits rapid phase advances via L-type VGCCs and an unknown signalling pathway. This quick adaptation of the clock to a light signal is referred to as clock resetting, as visualized in the PRC of mice (Fig. 2). However, of note is that the human PRC does not have a dead zone and hence is only similar but not identical to the mouse PRC^78^.

The PRC and the mechanism described above by which the clock responds to light are important for the short-term effects of light, mainly during the dark phase under a constant photoperiod (in the laboratory, 12 h of light and 12 h of darkness are commonly used). However, slow seasonal changes occur in environmental lighting throughout the year. As a result, the length of the light and dark (photoperiod) changes during the 24-h day, influencing the molecular clock. The clock is entrained to this change in photoperiod, inducing sustained plasticity in the SCN^79^. A recent study investigated the transcriptomic plasticity of the circadian clock in response to photoperiod^80^. Interestingly, no significant changes in the expression levels of core clock genes were observed. However, prominent differences across photoperiods were found in genes that regulate SCN neuropeptide signalling and light responses. Within this study, molecular elements shown previously to be involved in photoperiodic plasticity in the SCN, such as GABA-A receptor signalling and chloride co-transporters NKCC1 and KCC2^81–83^, were observed to be important. This appears to be accompanied by light-dependent reprogramming of DNA methylation^84^, producing persistent alterations in the SCN and altering its acute response to light pulses^85–87^. Ultimately, this indicates that entrainment to the photoperiod may overlap mechanistically with the acute response of the clock to light pulses, as described previously. This is supported by the identification of acute light-responsive genes involved in photoperiod entrainment, including dual-specificity phosphatase 4 (*Dusp4*), ras related dexamethasone induced 1 (*Rasd1*), and GTP binding protein overexpressed in skeletal muscle (*Gem*)^80,88^, of which *Gem* gene induction is regulated by CDK5 in response to acute light^66^. These genes regulate or are regulated by the CREB-signalling pathway in the SCN and are probably important for the daily alignment of the clock to seasonal changes in photoperiod. An alternate light-inducible signalling pathway involving ERK signalling, culminates in FOS/JUN binding to AP-1 sites on target genes (Fig. 4)^80^. Dexras1 was shown to potentially act as a link between NMDA receptors and ERK activation as the light-induced activation of ERK signalling was attenuated in mice lacking Dexras1. Furthermore, NMDA produces phase shifts in SCN neuronal activity which can be blocked through the pharmacological blockage of G_i/o_, however, this effect is absent in Dexras1 knockouts, demonstrating a link between NMDA receptors, G_i/o_ and ERK^89^. While only a fraction of the light-regulated transcriptome can be linked to photoentrainment functionally, the role of a large amount of this transcriptome still remains unclear.

## Mood-related behaviours and light

Mood is an affective state influenced by many factors such as motivation, alertness, attention, and anxiety. The various components comprising mood can be regulated by neurotransmitters like dopamine, serotonin, and noradrenaline^90^, illustrating the complexity of mood states.

Photoperiod not only induces neuroplasticity in the circadian system as described above^91^, but it also produces neurotransmitter switching in rats exposed to either short- or long-day photoperiods^92^. Under a short photoperiod, hypothalamic GABAergic interneurons switch to dopamine (DA) neurons, and the ablation of these neurons increases anxiety and depression-like behaviour^93^. In contrast, long photoperiods have the opposite effect. This suggests that monoaminergic systems in the brain are altered by changes in photoperiod, resulting in changes in mood-related behaviours. The length of photoperiod is modulated by DA signalling in the NAc, affecting female and male mice differently^94^. This is of interest for specific forms of depression in humans, e.g. SAD, which has a higher prevalence in females compared to males. As mice and rats are nocturnal, while humans are diurnal, the responses to light in nocturnal versus diurnal species may be different. Indeed, while the molecular mechanisms governing pacemaker activity in the SCN is conserved in nocturnal and diurnal mammals^95^, downstream responses may differ. In support, the photoperiod affected the number of DA neurons in the grass rat, a diurnal rodent, in an opposite manner compared to mice^96^. Interestingly, a short photoperiod evoked depressive-like behaviour in these animals reminiscent of SAD in humans. Therefore, Costello et al.^97^, applied a light protocol similar to that used in bright light therapy (BLT) for humans to grass rats. The resulting higher wakefulness, increased nighttime sleep quality, and improved rhythm entrainment were comparable to those observed in humans^97^. Interestingly, a similar BLT treatment had positive effects in nocturnal mice subjected to the forced swim test, a proxy for motivation-related behaviour. Mice subjected to a 30-min light pulse, two hours before daily light onset (ZT22), showed increased motivation^98^, resembling the positive effects of BLT in humans. In conclusion, it appears that the initial light signalling pathway which evokes the positive effects of BLT is likely conserved between nocturnal and diurnal species. However, the interpretation of this signal may affect downstream pathways in an opposing manner (e.g. neurotransmitter regulation) to achieve identical behaviour in nocturnal and diurnal species. This has its prerequisite in the light-dependent delay or advance of clock phase, which is conserved in nocturnal and diurnal species^99^. Importantly, SCN clock gene expression has the same phase in diurnal and nocturnal animals^100^; however, outside the SCN^101^ and in peripheral tissues^102^, it is opposite. Therefore, it is likely that the differences between diurnal and nocturnal species are at the level of cellular and neuronal networks rather than at the level of clock genes. This view could be tested by studying a recently described mechanism in mice for adaptation to photoperiod^103^ using a diurnal animal such as the grass rat.

## Molecular mechanisms linking light, circadian rhythms and mood

As previously discussed, we see that light can affect circadian rhythms and mood. However, questions emerge as to whether light can affect both systems independently or whether they intersect at the molecular level with light regulating both pathways similarly. The third possibility would be that light affects the clock, which then regulates mood, resulting in mood being indirectly regulated by light via the clock.

The first evidence that clock components may be involved in mood regulation was obtained in the fruit fly when circadian clock genes were observed to be required for cocaine sensitization (*per*, *clk*, *cyc*, *dbt*, but not *tim*)^104^. Similar observations highlighted the involvement of period genes (*Per1* and *Per2*) in cocaine sensitization in mice^105^. The self-administration of psychoactive substances such as alcohol^106^ and fentanyl^107^ may influence and/or be influenced by the clock particularly via *Period* genes and ultimately influence mood-related systems. Indeed, *Per2* mutant mice show increased extracellular glutamate and increased alcohol intake, both of which are normalized following the administration of acamprosate, a drug used to prevent alcohol relapse^106^. Furthermore, fentanyl was shown to suppress light-mediated phase shifts and was accompanied by the absence of *Per1* gene induction in the hamster SCN^107^. Interestingly, fentanyl was shown to stimulate Cdk5/p35 activity and inhibit the PKA signalling pathway through the activation of opioid receptors^108^, consistent with the inhibitory effects of fentanyl on light-mediated *Per1* induction^107^ (Fig. 4). At the same time, the activated opioid receptors modulate MEK/ERK signalling through Cdk5/p35/25 activity^108^. This pathway has been shown to regulate light-mediated gene expression^109^ and clock resetting^110^. These observations suggest that psychoactive substances and light may modulate mood-related behaviours and clock resetting via similar signalling pathways involving clock components such as *Per1*. Recently, the beneficial effects of light on mood involving the *Per1* gene were described^98^. Specifically, light-mediated induction of *Per1* in the LHb/PHb was necessary, as specific deletion of *Per1* in the LHb/PHb abolished the beneficial effects of light. However, it is not clear if this effect is related to normal clock function or whether it is a *Per1*-specific function independent of the clock.

Associations between clock genes and mood-related disorders have been described in several human studies. Single nucleotide polymorphisms (SNPs) in clock genes are associated with affective disorders like SAD, including *CLOCK*^111^, *PER2, NPAS2, BMAL1*^112^, *PER3*^113^, and *CRY1* and *CRY2*^114^. However, such small-scale SNP studies in humans have recently been discredited as false positives, because genome wide association studies (GWAS) did not confirm any single clock gene variant to be genome-wide significant (reviewed in ref. ^115^). However, assembling large cohorts as used in GWAS, there is often loss of phenotypic detail. Therefore, molecular links relating the circadian clock to depressive behaviour in humans are scarce. To close this gap, mouse models with genetic disturbances in clock genes are used to investigate their involvement in mood disorders.

### General deletions and mutations of clock genes in the entire organism

Mice overexpressing a truncated form of the CLOCK protein caused by a *Clock* gene mutation (*Clock Δ19*)^116^, displays decreased sleep and anxiety, and increased reward-related behaviour^117^ (Table 1). Since overexpression of the mutated protein may affect many cellular processes in a non-specific manner, it is unclear whether these observations are caused directly by the circadian clock or are of an indirect nature. Interestingly, deleting the *Clock* homologue *Npas2*^118^ reduced anxiety similarly to the *Clock Δ19* mutation^119^. Furthermore, mice that express an unstable non-nuclear protein due to a mutation in the clock component *Per2* (*Per2*^*Brdm1*^)^120,121^, show decreased despair^122^, similar to *Clock Δ19* mutated mice (Table 1). In both mutations (*Clock Δ19* and *Per2*^*Brdm1*^), the mesolimbic dopaminergic system is affected, potentially due to changes in dopamine-synthesizing^123^ or degrading enzymes^122^. In contrast to the decreased despair phenotype of *Per2*^*Brdm1*^ mice, *Per1* knock-out mice show increased despair-like behaviour, while animals with deletions in both *Per1* and *Per2* genes show increased anxiety^124^, comparable to the *Per1* knock-out animals^98^ (Table 1). Similarly, mice with deletions in *Cry1*, *Cry2* or both, displayed changes in anxiety; however, despair-related behaviour was unaffected^125–128^ (Table 1). Interestingly, restoring circadian rhythmicity in *Cry1/Cry2* double knock-out (KO) mice by rescuing *Cry1* expression in the SCN improved anxiety-like behaviour as well as metabolic deficits^129^. Discrepancies between studies suggest that the genetic differences between mouse strains used, breeding conditions, and/or variations in animal facilities may influence behaviour. Particularly, this may be the case for behaviours sensitive to environmental or housing conditions, including tests used to measure depressive- or anxiety-like behaviour in mice. Indeed, significant differences in exploratory behaviour (and thus anxiety-like behaviour) is observed between males and female mice in the open field, with group-housed females spending more time in the open area than their individually-housed counterparts. This observation is inverted in male mice, with individually-housed males spending significantly more time in the open section than their group-housed counter parts^130^. Thus, the sex used and the way animals are housed can greatly influence the observations between studies.

**Table 1:** Mood related phenotypes of mice lacking clock genes in all cells or specific cell types and/or brain regions (adapted from Imamura and Takumi^152^)

Genotype	Circadian period	Phenotype	Reference
*Bmal1* KD in SCN glia-specific *Bmal1* KO	Unknown Unknown	Increased depression-like behaviour, despair, increased anxiety-like behaviour No effect on mood related behaviour	Landgraf et al.^144^ Martini et al.^145^
*Chrono* KO	Long	Increased glucocorticoid levels in response to stress	Goriki et al.^138^
*Clock* mutant (*Clock* Δ19) CKI inhibition in *Clock* Δ19 *Clock* KD in VTA	Long Unknown Unknown	Hyperactivity, decreased sleep, decreased depression-like behaviour (mania), decreased anxiety, increased reward seeking for cocaine and sucrose Reversal of the anxiety-related behaviour, partial reversal of depression-related phenotypes Hyperactivity, increased manic-like state of less anxiety, increased depression-like behaviour	Roybal et al.^117^ Arey and McClung, 2012^143^ Mukherjee et al.^148^
*Cry1* KO *Cry2* KO *Cry1/2* double KO *Cry1/2* double KO *Cry1/2* double KO *Cry2* KO *Cry1/2* double KD in D1R-MSN	Short Long Arrhythmic Arrhythmic Arrhythmic Long Unknown	Increased anxiety-like behaviour Unaffected depression-related behaviour Increased anhedonia Unaffected despair behaviour Limited ability to habituate to new environments, no differences in anxiety or depression-related behaviours Decreased despair-like behaviour, increased anhedonia, unaffected anxiety-like behaviour Decreased susceptibility to stress-induced helplessness, increased NAc neuronal activation at night	De Bundel et al.^125^ Savalli et al.^126^ Huhne et al.^127^ Sokolowska et al.^128^ Porcu et al.^92^
*Fbxl3*^*Afh/Afh*^	Long	Decreased anxiety-like behaviour, decreased depression-like behaviour	Keers et al.^142^
*Npas2* KO *Npas2* KO	Short Short	Impaired cued and contextual memory Reduced anxiety-like behaviour, GABAergic neurotransmission in the VTA is affected	Garcia et al.^118^ Ozburn et al.^119^
*Per2* mutant (*Per2*^*Brdm1*^) *Per1/2* double KO (*Per1*^*Idc*^*/Per2*^*Idc*^) *Per1/2* double KD in NAc glia-specific *Per2* KO neuron-specific *Per2* KO glia-specific *Per2* KO in NAc *Per1* KO LHb-specific *Per1* KO	Short Arrhythmic Unknown Normal Normal Normal Short Unknown	Depression-resistant-like behaviour (mania-like), reduced expression and activity of MAOA, increased dopamine levels in the ventral striatum Increased anxiety-like behaviour Increased depression-like behaviour Altered despair and anxiety-like behaviour (females) Altered despair but not anxiety-like behaviour Altered despair but not anxiety-like behaviour Increased depression-like behaviour (in females, weak in males) No effect on mood-related behaviour, but beneficial effects of nocturnal light at ZT22 on despair are abolished (females)	Hampp et al.^122^ Spencer et al.^124^ Martini et al.^145^ Olejniczak et al.^98^
*Rev-erbα* KO *Rev-erbα* KD in NAc	Short Unknown	Increased mania-like behaviour, increased expression of tyrosine hydroxylase (TH), increased dopamine Increased sociability, reduced anxiety-like behaviour, unaffected depression-like behaviour (female mice), no significant behavioural effects in male mice.	Chung et al.^131^ Zhao et al.^149^

**Fig. 5:**
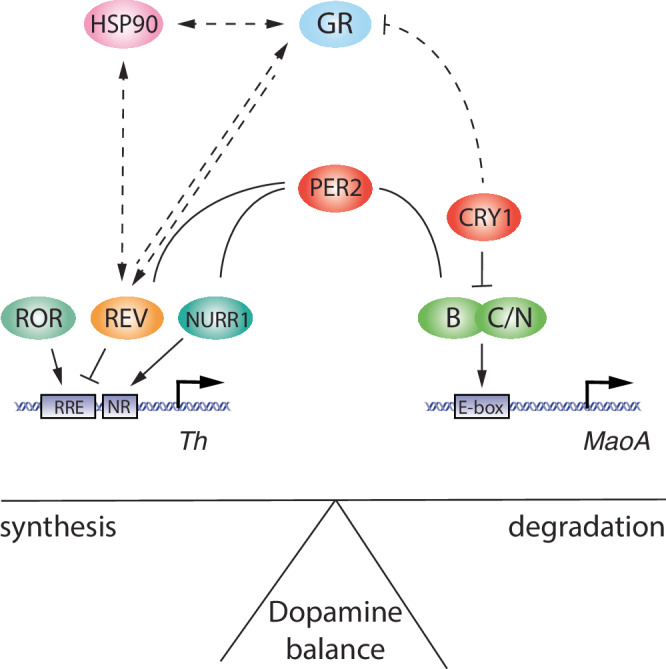
Model for balancing dopamine levels by clock components. The left part of the model shows the transcriptional regulation of *tyrosine hydroxylase gene* (*Th*), encoding the rate-limiting enzyme of dopamine synthesis. *Th* is regulated by the nuclear receptors ROR, REV-ERB, and NURR1 via the regulatory promoter elements RRE and NR. The right part of the model depicts the regulation of the *monoamine oxidase A gene* (*MaoA*), encoding the rate-limiting enzyme of dopamine degradation. *MaoA* is regulated by BMAL1 (B), CLOCK (C)/NPAS2 (N) via an E-box element present in the *MaoA* promoter. PER2 can influence both arms of dopamine regulatory genes via interaction with REV-ERB/NURR1 and interference with BMAL1/NPAS2. The hatched lines illustrate a regulatory pathway for REV-ERB present in the liver, involving heat-shock protein 90 (HSP90) and glucocorticoid receptor (GR). This pathway may potentially also be present in the brain.

The nuclear receptor REV-ERBα, a negative regulator of *Bmal1*, *Clock* and *Npas2* (Fig. 3), is implicated in the regulation of mood-related behaviours in mice. Animals with a deletion in the *Rev-erbα* gene show a similar phenotype to *Per2*^*Brdm1*^ animals, with a notable elevation in striatum dopamine levels caused by increased transcriptional levels of the tyrosine hydroxylase gene^131^ (Table 1), which encodes the rate-limiting enzyme in dopamine synthesis. As *Per2* regulates the rate-limiting enzyme of dopamine degradation, momoamine-oxidase A^122^, it appears that both clock components REV-ERBα and PER2 balance the synthesis and degradation of dopamine, thereby influencing mood (Fig. 5). Because PER2 protein was shown to interact with nuclear receptors, including REV-ERBα and NURR1^132^, it can be hypothesized that the level of PER2 protein may modulate temporal synchronization between the clock and the regulation of ccgs as shown in Fig. 5. However, REV-ERBα may not only influence the dopaminergic system but also the serotonergic system, as has been proposed recently^133,134^.

The development of mood disorders is likely associated with changes in glucocorticoid levels influenced by stress. Glucocorticoids act by binding to glucocorticoid receptors (GR), and the subsequent translocation of GRs to the nucleus activates the transcription of target genes. Importantly, this process is influenced by REV-ERBα, which competes with GRs for the binding of heat-shock protein (HSP) 90 in the cytoplasm. Thus, these two nuclear receptors influence each other’s stability and nuclear localization in an opposing cyclic manner^135^ (Fig. 5). This is paralleled by the opposing rhythmicity of REV-ERBα and GR target gene expression^136,137^. However, these observations were made in liver. It remains to be seen whether the same mechanism applies to brain tissue. Furthermore, *Chrono*, a less well-known negative regulator of the circadian clock, appears to be involved in glucocorticoid signalling, as the lack of this gene resulted in higher corticosterone levels following restraint stress when compared to control animals also subjected to this stressor^138^ (Table 1). Hence, clock components such as REV-ERBα and *Chrono* may affect mood via noradrenergic signalling. Although it remains to be tested whether *Chrono* KO mice display changes in mood-related behaviours.

As shown in Fig. 3, clock components are regulated by post-translational modifications (PTMs). Therefore, it is not surprising that post-translational modifiers such as FBXL3 or CDK5, which phosphorylate clock components, can modulate mood-related behaviours^139–143^ (Table 1). Taken together, there is good evidence that clock components directly or indirectly modulate pathways involved in mood regulation. Taking caution to conclude that the circadian clock regulates mood is warranted. The evidence described above is based on clock gene deletions that manifest during embryogenesis, and these deletions are in the entire organism. Hence, in mice lacking specific clock genes in the entire organism, a mixture of developmental and systemic effects, including sleep may impact on mood-related behaviours.

### Localized deletion or suppression of clock genes in the brain

Since light information is transmitted to specific brain regions to affect certain clock genes and mood, it is of great interest to understand how clock genes and lack of them in specific brain areas would affect mood. Several laboratories have started to investigate this question by using either genetically modified mice that allow the deletion of a specific gene in specific cell types or by using viral mediated transfection systems that allow the deletion of genes in specific brain areas of the adult animal.

*Bmal1* is considered the most important clock gene, because its absence leads to arrhythmic activity behaviour, indicating a non-functional clock. Therefore, knock-down (KD) of the *Bmal1* gene through RNA interference in the SCN was studied. While SCN-*Bmal1*-KD animals showed circadian activity rhythms with a longer period compared to controls, these mutants displayed several characteristics of depression-related behaviours^144^ (Table 1). This suggests that the clock component *Bmal1* affects mood-related behaviour including despair. However, this is not necessarily related to the circadian clock, because these mice were not arrhythmic. Interestingly, lack of *Bmal1* in glial cells did not affect despair^145,146^, indicating that the despair-related phenotype observed by Landgraf et al.^144^ is relevant to SCN neuronal populations. The deletion of *Bmal1* in CaMK2a glutamatergic neurons of the mouse mPFC (Fig. 1) was associated with changes in behaviour as assessed in a chronic despair model^147^, illustrating how the deletion of a clock component in a specific brain area can have specific effects.

Knock-down of the *Clock* gene in the VTA resulted in hyperactivity, less anxiety and increased depression-like behaviour^148^ (Table 1). While hyperactivity and decreased anxiety have been observed in the *Clock Δ19* mutant as well, depression-like behaviour was decreased in these animals^117^, which is just the opposite of the observation in the VTA specific KD of *Clock*. The reason for this is manyfold. First, *Clock Δ19* mutant mice overexpress a mutant form of *Clock* (it is not a deletion of *Clock*), and therefore may affect cellular processes in an unspecific manner. Second, the mutation is present in all cells and not only in the VTA, and hence presence of mutant *Clock* in other tissues may contribute to the phenotype observed in *Clock Δ19* mutant animals. This underscores the importance of studying gene deletions in a brain region specific manner.

Mice with a KD of *Per1* and *Per2* genes in the NAc displayed increased anxiety, as observed in the whole-body *Per1/2* KO animals^124^ (Table 1). This implicates NAc *Per* gene expression in the regulation of anxiety stress responses. Interestingly, however, deletion of *Per2* in glial cells of the NAc did not affect anxiety, but significantly impacted despair^145^, suggesting that the lack of glial *Per2* in the NAc does not contribute to anxiety. This illustrates the potential of deleting clock genes in specific cell types and brain areas to understand the relationships between clock gene expression and mood regulation.

Suppression (KD) of *Rev-erbα* in the NAc increased sociability and reduced anxiety-like behaviour but had no effect on depressive-like behaviour in female mice. In contrast, male mice did not show alterations in any of the behaviours tested^149^, comparable to observations made in *Per2* KO animals, whereby only female mice displayed strong phenotypes^98^ (Table 1). These observations suggest that *Rev-erbα* in the NAc alone is not sufficient for the regulation of depressive-like behaviour. However, this needs to be experimentally tested by thoroughly deleting *Rev-erbα* in the NAc, because KD only reduces but does not completely eliminate expression of *Rev-erbα* mRNA.

The specific deletion of cryptochrome genes *Cry1* and *Cry2* in dopamine 1 receptor-expressing medium spiny neurons of the NAc^92^, resulted in mice displaying reduced susceptibility to stress-induced helplessness and increased neuronal activation in the NAc at night. This suggests a role for CRY in the regulation of the dopamine reward system.

As highlighted earlier, the LHb is an important brain region involved in the regulation of depression-related behaviours in mice^25^. This brain region appears to be important for the beneficial effects of light on mood, because the lack of *Per1* in the LHb abolishes the beneficial effects of light^98^ (Table 1). However, mice lacking *Per1* in the LHb do not display a depression-like phenotype compared to control animals^98^, suggesting that *Per1* in the LHb influences only the beneficial effects of light but is not involved in regulating mood in the absence of light used in bright light therapy. *Per1* expression in other brain regions such as the NAc or VTA are potential candidates whereby mood can be affected directly. However, this requires clarification in future experiments using mice with specific deletions in these specific brain regions.

Overall, it appears that clock genes in specific brain regions and cell types can affect mood-related behaviour. While we know that light can affect mood by stimulating specific brain regions, including the induction of *Per1* gene expression in the LHb, further studies are required to fully dissect the influence of light in brain regions implicated in mood and how light impacts clock genes in these brain areas.

## Conclusions and outlook

Light has a profound influence on the circadian clock and mood, not only in rodents but in humans as well^150^. Depending on the time when light hits the retina, different behavioural outcomes can be observed. This is visualized by the phase response curve (PRC) where light perceived early in the prospective dark phase elicits phase delays but when perceived in the late part of the dark phase it elicits the opposite response, namely phase advances (Fig. 2). Interestingly, a similar relationship between the timing of light and its effects on mood-related behaviours have been observed. Light in the early part of the dark phase appears to be pro-depressive^26^, whereas later in the dark phase it seems to be anti-depressive^7,98^. Therefore, it is intriguing to speculate that there may be a PRC for the relationship between light and mood-related behaviours. This potential mood PRC could possibly be used to develop treatments for neurobiological disorders such as depression.

The main gap in knowledge lies in understanding the molecular pathways triggered by light and how exactly these connect to the cells regulating neurochemical pathways. Light detection and signalling is a very dynamic process involving immediate effects (Fig. 4, delay or advance of clock phase) that later turn into a medium-term change in cellular signalling and metabolism (photoperiod adaptation, change of neurotransmitter signalling). How exactly light influences the balance (synthesis vs degradation) of dopamine metabolism (Fig. 5) is not understood. The core clock genes affected by light are the *Per* genes, particularly *Per1*. Therefore, an immediate change in their expression levels induced by light may have a downstream effect on *Ccgs*, which regulate key biochemical pathways. One scenario could be that protein levels of PER1 and PER2 and their respective ratio would determine which Ccgs are induced or repressed and hence which biochemical pathways are activated or repressed, thereby regulating physiological/behavioural outcomes. Furthermore, mutations in clock genes impact on nonvisual photoreception in the mouse^151^ and may by this avenue affect various behaviours modulated by light.

Our overall knowledge of specific aspects of light on behaviour is relatively well investigated. However, the molecular underpinnings are not understood. We have fragmentary knowledge of specific molecular/cellular processes of light on signalling pathways and the circadian clock but do not understand how these pathways are molecularly linked and what the chain of events is to generate the observed behavioural consequences initiated by light treatment.

## Data Availability

No datasets were generated or analysed during the current study.
